# Tricuspid Regurgitation in Left Ventricular Systolic Dysfunction: Marker or Target?

**DOI:** 10.3389/fcvm.2021.702589

**Published:** 2021-06-28

**Authors:** Davide Margonato, Francesco Ancona, Giacomo Ingallina, Francesco Melillo, Stefano Stella, Federico Biondi, Antonio Boccellino, Cosmo Godino, Alberto Margonato, Eustachio Agricola

**Affiliations:** ^1^Echocardiography Laboratory, Istituto di Ricerca a Cura e Carattere Scientifico (IRCCS) San Raffaele Scientific Institute, Milan, Italy; ^2^Cardiology Department, University of Pavia, Pavia, Italy; ^3^Department of Clinical Cardiology, Istituto di Ricerca a Cura e Carattere Scientifico (IRCCS) San Raffaele Scientific Institute, Milan, Italy

**Keywords:** tricuspid regurgitation, left ventricular dysfunction, heart failure, echocardiography, right heart failure

## Abstract

Far from being historically considered a primary healthcare problem, tricuspid regurgitation (TR) has recently gained much attention from the scientific community. In fact, in the last years, robust evidence has emerged regarding the epidemiological impact of TR, whose prevalence seems to be similar to that of other valvulopathies, such as aortic stenosis, with an estimated up to 4% of people >75 years affected by at least moderate TR in the United States, and up to 23% among patients suffering from heart failure with reduced ejection fraction. This recurrent coexistence of left ventricular systolic dysfunction (LVSD) and TR is not surprising, considered the multiple etiologies of tricuspid valve disease. TR can complicate heart failure mostly as a functional disease, because of pulmonary hypertension (PH), subsequent to elevated left ventricular end-diastolic pressure, leading to right ventricular dilatation, and valve tethering. Moreover, the so-called “functional isolated” TR can occur, in the absence of PH, as a result of right atrial dilatation associated with atrial fibrillation, a common finding in patients with LVSD. Finally, TR can result as a iatrogenic consequence of transvalvular lead insertion, another frequent scenario in this cohort of patients. Nonetheless, despite the significant coincidence of these two conditions, their mutual relation, and the independent prognostic role of TR is still a matter of debate. Whether significant TR is just a marker for advanced left-heart disease, or a crucial potential therapeutical target, remains unclear. Aim of the authors in this review is to present an update concerning the epidemiological features and the clinical burden of TR in the context of LVSD, its prognostic value, and the potential benefit for early tricuspid intervention in patients affected by contemporary TR and LVSD.

## Introduction

The long-time accepted idea that tricuspid regurgitation (TR) is a benign valvular condition has been deeply rebutted by almost one decade of insights into its epidemiological and clinical implication. While trivial TR is a common finding during routine echocardiography examination of asymptomatic subjects and is considered almost a physiological condition (1), most recent data suggest that at least moderate TR is a frequent condition too, worsening mid and long-term survival, particularly in patients >75 years old, and in those suffering from left ventricular systolic dysfunction (LVSD) (2–5). TR coexisting with LVSD has been the most investigated across all TR subtypes. Recent studies, through the significant help of modern echocardiographic techniques, have allowed us to obtain a more detailed evaluation of the many different possible abnormalities of the TV apparatus in case of concomitant LVSD, and of their consequences. However, in spite of the increasing knowledge on the pathological implications of significant TR, its independent prognostic role, the most appropriate type and time of treatment are still heavily debated.

The current review analyzes the prevalence, the morphological types of TR, and its prognostic value in the context of LVSD.

## Epidemiology

At least trivial TR is a frequent finding during routine echocardiographic evaluation (1, 6). This has led to the trusted concept that TR is a relative benign condition: as a direct consequence, there has historically been a lack of epidemiological data regarding the prevalence of TR, both in the general population and in those suffering from left-heart disease. Nonetheless, in the recent years, following the dramatic growing attention to tricuspid valve pathology, several studies, with reliable systemic echocardiographic evaluation, have focused on the frequency of hemodynamically relevant TR. In the Olmsted County community (7), the prevalence of all-cause > moderate TR, adjusted for the age, and sex distribution of the United States white population, was of 0.55% [95%, confidence interval (C.I.) 0.50–0.60]. The prevalence was higher in women (*p* < 0.01) and strongly linked to age (*p* < 0.0001), reaching up to 4% in people older than 75; interestingly, LVSD accounted for 12.9% of all TR causes. The prevalence of significant (graded >2/4 on Color Doppler evaluation) TR was 10.2% among 2,054 consecutive patients with different types of cardiac pathologies evaluated over a 3-month period (8). In the context of LVSD, the presence of TR was greatly associated with non-ischemic cardiomyopathy [odds ratio (OR) 6.2 (1.8–21.3); *p* = 0.004], ischemic cardiomyopathy [OR 5.6 (1.5–21.8); *p* = 0.012], and heart transplantation [OR 10.4 (3.4–31.8); *p* < 0.001). In a retrospective analysis performed on 6,309 consecutive patients undergoing echocardiography in a single tertiary center in Milan, Italy (9), 10.9% of patients suffered from at least moderate TR: patients with severe TR presented worse New York Heart Association functional class (III or IV, 19 vs. 40%; *p* = 0.005), more signs and symptoms of right ventricular failure (15 vs. 40%; *p* = 0.0001), and had a lower left ventricular ejection fraction (LVEF) (52.8 ± 14 vs. 50 ± 15; *p* = 0.022). A national United Kingdom cohort (10) reported that TR, at the time of echocardiographic evaluation for suspected heart failure (HF), was the most common observed valvular disease (5% prevalence of moderate or severe TR). In the largest study to date evaluating the impact of functional TR in a cohort of 13,026 patients affected by HF with reduced LVEF (3), the prevalence of moderate or severe TR was 23%; again, compared to patients with milder grade of TR, those affected by moderate or severe functional TR presented with more severe LVSD (*p* < 0.0001).

If we agree with Enriquez-Sarano et al. (2) that the definition of public health crisis relies on a tryptic association based upon the frequency of the condition, its impact on the outcome and the limited treatment received by those affected by this condition, these available epidemiological data strongly support the opinion of TR being a “public health crisis,” particularly in the setting of concomitant LVSD. Therefore, indeed TR is a frequent pathological condition, with a vast majority of patients affected by moderate and severe TR who will only receive medical therapy during their lifetime; the epidemiologic burden of TR with regard to LVSD is a direct consequence of the different underlying abnormalities that might involve the tricuspid valve (TV) in this clinical scenario.

## Etiologies and Mechanisms of TR in LVSD

**Figure 1:**
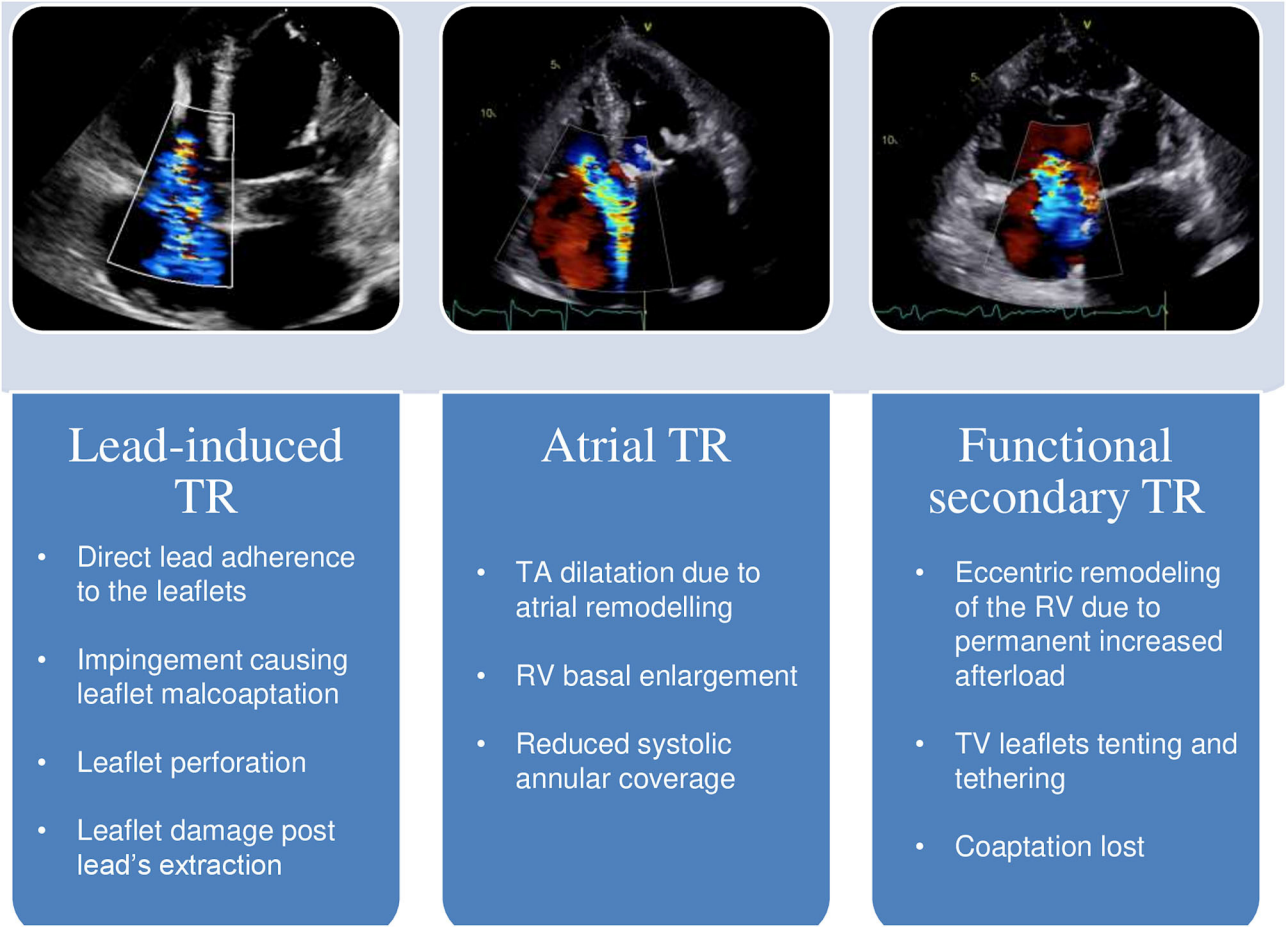
Different mechanisms of TR in LV systolic dysfunction. In the panels above, transthoracic echocardiography color-doppler images; below, the main anatomic features of each related subtype. TA, tricuspid annulus; RV, right ventricle; TV, tricuspid valve; TR, tricuspid regurgitation; LV, left ventricle.

Organic TR, acquired or congenital, is caused by a pathological process affecting any of the elements of the TV apparatus. Lead-induced TR is by far the most common subtype of organic TR among patients with LVSD (Figure 1). The prevalence of this type of TR is still a matter of debate, but the reported frequency of TR following lead implantation ranges from 7 up to 45% (11–13). These conflicting reports are not surprising, as many studies are based on 2D transthoracic echocardiography evaluation of the TV, which presents severe limitations on the assessment of the TV apparatus, on the identification of the lead crossing the annulus and on the specific mechanism leading to lead-induced TR (12, 14, 15). The diagnostic complexity of appropriate lead-induced TR evaluation is of critical relevance: in fact, TR after lead's implantation is not always organic, as it may even result from the progression of the underlying left-heart disease or from a pacing-induced alteration of the right ventricle (RV) geometry (16). 3D echocardiography, transthoracic as well as transesophageal overcome the limitations of the 2D method, allowing a precise visualization of the TV apparatus and of the leads from both the atrial, and the ventricular perspective, a significant help in the evaluation of the underlying mechanism of TR (14, 17, 18). Lead-induced TR can be the final common result of different processes: direct lead adherence to the leaflet or to the subvalvular structures, impingement causing malcoaptation of the leaflets, leaflet perforation, or direct damage of the TV apparatus after lead's extraction (14, 17, 19, 20). Mobile leads across the center of the valve or placed within the commissures appear as the most appropriate positions in order to avoid post-implantation significant TR (14, 17). Whether the number of leads crossing the TV annulus, the position of the leads within the RV or the degree of right ventricular pacing are correlated to a more severe presentation of TR, remains unclear (16, 21–24). The presence of the leads is in itself associated with the risk of device endocarditis, predisposing the TV apparatus to a direct damage (25). Lead's infection has a significantly high mortality rate when the TV apparatus is involved (26), and is frequently managed with lead's extraction, further increasing the potential harm to the TV. Despite most of the studies on these subtype of TR don't differ between pacemaker (PM) and implantable cardioverter-defibrillator (ICD), there are inherent differences related to the presence of an ICD: compared to PM, ICD is often implanted in patients with LVSD, raising the suspect of differential diagnosis with functional TR, and ICD coils present greater stiffness and thickness compared to PM wires, incrementing the risk of interference with the TV apparatus, and of weakening the color doppler signal. Among organic causes of TR, flail leaflets needs a special mention. It has a wide range of causes, in particular post-traumatic, caused by endocarditis, following leads extraction, or is a result of a pure myxomatous degenerated valve (27).

Functional TR is the most common subtype, accounting for up to 85% of all TR cases (28), and is caused by functional changes in TV apparatus in the setting of concomitant RV remodeling, most frequently from PH due to left-heart disease. Functional TR can be present in the absence of PH (29), and growing evidence regarding its link with atrial fibrillation (AF) is emerging (Figure 1). AF is the most common arrhythmia in HF, shares with LVSD many predisposing risk factors, and an increased prevalence in the elderly, and most important, often cooperates with it to sustain each other in a vicious cycle (30, 31). Although AF has been frequently related to isolated functional TR (32, 33), lately it has been proved to be strongly and independently linked to the presence of more severe FTR in HF patients with reduced LVEF, even after multivariate analysis taking PH into account (3). The leading mechanism of functional atrial TR is an enlargement of both the tricuspid annulus and the RV basal diameter (RV conical shape) with normal leaflets length, reduced systolic annular coverage in the absence of significant valve tethering. Again, 3D echocardiography facilitates an accurate evaluation of these specific morphologic abnormalities affecting the TV apparatus by precisely measuring the tethering height, area and volume, and the TV annulus area (34). Therefore, patients with AF and LVSD, in particular if elderly, might be recognized as a high-risk category for functional TR, even in the absence of PH.

Mitral regurgitation [MR] (4, 35), severe aortic stenosis (36), and LVSD (37) are the main causes of functional TR. In patients surgically treated for MR at least moderate and clinically severe TR have been reported in up to 37 and 70%, respectively (35), while in patients with severe AS the long-term prevalence of moderate or more TR is around 25% (36). It is interesting to underline that the cardiac damage caused by AS-related pressure overload may not always be sequential, i.e., from a hypertrophied LV with increased filling pressure to PH, RV remodeling and TR, but genetic predisposition and individual susceptibility may play an important role, although the natural evolution of AS-related cardiac injury still needs to be fully clarified (36, 38). Chronically elevated left-ventricle filling pressure frequently results in the development of PH and subsequent RV structural abnormalities (39, 40) (Figure 1). In fact, an increase in the RV afterload initially spurs compensatory remodeling of the myocardium; however, permanent afterload increase promotes a RV decompensated phenotype (41), the leading cause of TV remodeling in functional TR. In particular, the elongation and eccentricity of the remodeled RV account for tricuspid papillary muscles lateral and apical displacement, TV leaflets tenting and tethering and eventual coaptation lost, despite the absence of significant annular enlargement (42, 43). PH can be subdivided between precapillary and post-capillary depending on the component of the affected pulmonary circulation. Precapillary PH [pulmonary artery wedge pressure (PAWP) ≤ 15 mmHg] is caused by arterial remodeling with increased pulmonary vascular resistance; post-capillary PH (PAWP > 15 mmHg) is related to an increase in pulmonary venous pressure among patents affected by left-sided heart diseases (44). PH causes an increase afterload of RV. Acute increase of RV afterload, like in pulmonary thromboembolism, is associated with RV dilatation due to its thinner wall, and lower volume-to-wall-surface area ratio (44). In the setting of chronic afterload, the initial adaptation of RV is characterized by quite preserved volumes and function with wall hypertrophy to match afterload (44). Afterwards, chronic pressure overload induces a progressive RV dilatation with increased filling pression. RV eccentric hypertrophy maintains the appropriate ventricular-vascular coupling inducing TR through annular dilatation and TV remodeling and increased metabolic demand. This further RV remodeling leads to RV failure and clinically decompensated HF. This remodeling of the RV ventricle presents a direct therapeutical relevance, as TV leaflet tethering distance and area predicts significant residual TR after TV annuloplasty. Therefore, in patients with severe TV remodeling, annuloplasty is not the surgical therapy of choice (45).

It is mandatory to underline that functional TR can persist or even progress despite appropriate pharmacological treatment or interventional resolution of the concomitant left-heart disease (37, 46, 47).

Considering the extreme heterogeneity of the “TR population” and the natural progressive history of this disease, a correct evaluation of the morphologic type of TR, focusing on the annular dimensions, the subvalvular apparatus, the tenting area, and the RV function and dimension, is pivotal at the time of TR diagnosis. Moreover, we should even aim to assess the independent impact on the outcome of each subtype of TR, as different subtypes and different stages of significant TR may imply different treatment options, varying from isolated TR surgery, and transcatheter options to palliative procedures only in selected patients.

## The Pathophysiology of Organ Impairment in TR and LVSD

Although the question concerning the prognostic role of TR in the natural history of LVSD has been debated for decades, only in the recent years we have gained a significant amount of evidences, with well-designed studies across most of the various clinical scenarios (i.e., organic, functional, isolated, or in the context of multivalvular heart disease), that could help dealing with our initial dilemma: does TR represent just a marker for advanced myocardial disease, or is it an independent cause of the adverse outcome and a potential therapeutic target? The answer may not be univocal. Indeed, various features associated with a greater severity of TR, such as LVSD, PH, and AF, are all independently associated with a decreased long-term survival; nonetheless, in case of LVSD, different plausible mechanisms could directly and indirectly relate TR to a poor outcome.

First, albeit volume overload is initially well-tolerated by the RV, compared to pressure-overload (48), if sustained over time it can induce dramatic repercussions. In fact, chronic volume-overload, induced by severe TR, promotes an increase in RV end-diastolic volume, preload and wall-tension, resulting in RV ischemia and, accordingly, RV systolic disfunction and increased overall mortality (49). Another critical direct consequence of right-heart volume overload is the occurrence or worsening of simultaneous LVSD, following leftward interventricular septal displacement and the subsequent reduction in left ventricle preload and increase in left-ventricle end-diastolic pressures (49, 50); moreover, significant TR reduces RV stroke volume and, therefore, left ventricle preload and cardiac output (50). Elevated right-atrial pressure caused by TR can lead to atrial remodeling and to the development of supraventricular tachyarrhythmias, compromising cardiac stability and prognosis of patients with LVSD (51).

Notably, hemodynamically relevant TR is a primary effector mechanism for the increase in central venous pressure (CVP). Systemic venous congestion is a main determinant of reduced renal blood flow and, subsequently, of the decline of glomerular filtration rate and of the exhaustion of renal autoregulatory capacity (52–54). A pathological rise in renal venous pressure is an independent risk factor for renal decreased function in patients with HF and, therefore, for adverse outcome, even in the absence of impaired cardiac output, another mechanism by which TR may reduce renal blood flow (55–57).

Hepatic failure, resulting from both hepatic congestion and reduced hepatic perfusion, is crucially combined to TR severity (58). As RV pressure is transmitted straight to the hepatic veins, TR is particularly susceptible to result in severe passive congestion (59). This increase in CVP caused by severe TR leads to atrophy of the hepatocytes and sinusoidal edema that can directly affect oxygen diffusion to the hepatocyte (60). Hepatic failure usually is revealed by an increment of the markers of cholestasis, rather than transaminases, another factor independently associated with mortality among patients with LVSD (61), and by a reduction in albumin synthesis, leading to a vicious cycle that sustains the increase of hydrostatic pressure and abdominal edema. Finally, the pathological augmentation of CVP may be primarily responsible for a compromised gastrointestinal function, a typical occurrence in the advanced stages of HF (62): visceral edema and intra-abdominal hypertension can lead to adverse sequelae such as malnutrition through reduced nutrient absorption (63), protein-losing enteropathy (64), bacterial translocation from the intestinal gut (65), and diuretic malabsorption and resistance (66).

**Figure 2:**
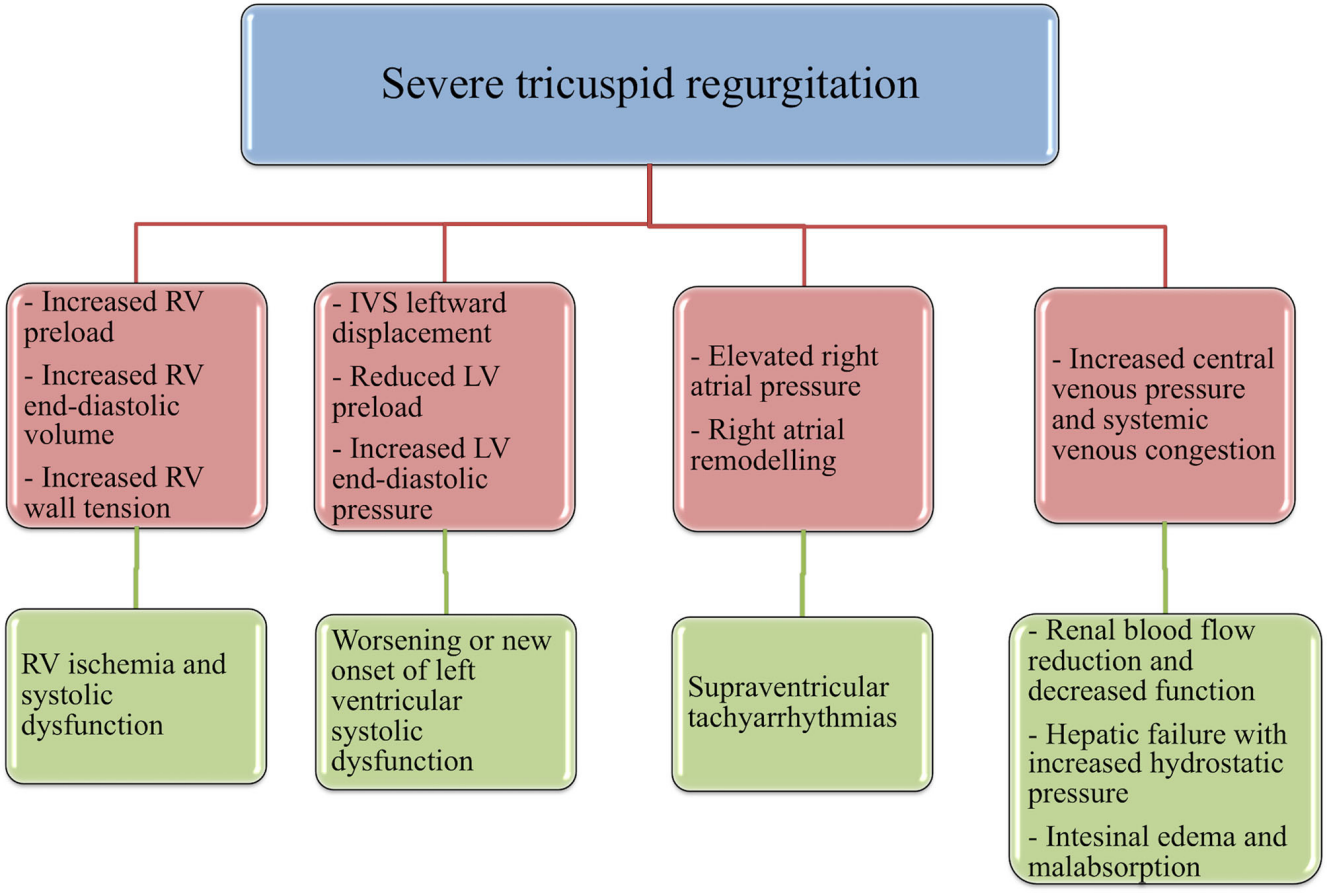
Different pathophysiological mechanisms that may relate tricuspid regurgitation to an independent prognostic role in the context of LV systolic dysfunction. RV, right ventricle; LV, left ventricle.

In summary, several TR-induced mechanisms can affect the prognosis of these patients by both reducing left-ventricle pump function and leading to multiple organs dysfunction in the context of right-heart failure (Figure 2). We must improve our ability to recognize and manage the above-mentioned conditions, as time is crucial to avoid a stage of end-organ damage that would waste our effort in treating these patients.

## The Prognostic Impact of TR: A Reappraisal

Despite different pathophysiological implications by which TR, both itself and indirectly, can contribute to a poor prognosis, uncertainty has long persisted regarding its independent association with negative outcomes in patients with LVSD. Four main issues need to be addressed to evaluate the role of TR in LVSD. First, the hypothesis of severe functional TR as a marker of end-stage myocardial disease is strongly supported by the pathophysiological implications of LVSD, particularly if corroborated by left-valvular disease, PH and AF, and by the amount of evidence emerged regarding the poor prognosis of these patients. Second, although it is unanimously accepted that, in the vicious cycle of a damaged left ventricle affected by systolic dysfunction, leading to PH and RV maladaptive remodeling, the appearance of relevant TR warrants a state of poor long-term survival, to demonstrate its prognostic role regardless of the multiple co-existing cardiac comorbidities can be extremely challenging. Third, one could argue that the impact of TR in this context may depend upon its grade or the severity of LVSD and PH: this has a therapeutical relevance, since we lack knowledge on the appropriate timing of intervention, and different subgroups of patients would benefit more than others from invasive treatment of TR. In particular, invasive hemodynamic assessment through right heart catheterization provide us fundamental and often underestimated parameters, such as cardiac index, pulmonary wedge pressure, the subgroup of PH and its eventual reversibility, that may indeed help us to correctly identify the candidate patient that would benefit most from TR invasive treatment (67).

Finally, if considered a therapeutical target, we eagerly await for randomized prospective studies demonstrating a safety and beneficial effect of isolated TR correction in patients with LVSD, in order to definitely clarify its clinical role.

In the recent years the growing interest from the scientific community has led to a “Copernican Revolution” on the vision of TR-related poor outcome and on the need for an appropriate therapeutical management, a reappraisal that could bring us at least closer to an answer for our initial question (see Table 1).

**Table 1:** Summary of the main studies cited in the text on the prognostic role of TR with LV systolic dysfunction (specific references from the text are mentioned).

**References**	**Year**	***n*^**°**^ patients**	**Patient population**	**Results (see the text for HR and *p*-values)**	**Comments**
Benfari et al. (3)	2019	11,507	At least trivial FTR in an HFrEF (EF < 50%) cohort	5 years survival reduced with increasing severity of FTR, independently of baseline characteristics	Largest study to date on FTR in HF
Topilsky et al. (68)	2018	291	At least trivial FTR in an HFrEF (EF < 50%) cohort	Severe TR (EROA > 0.4 cm^2^) associated with increased mortality, even after comprehensive adjustment	First study to link the threshold of EROA > 0.4 cm^2^ to survival in patients with systolic dysfunction
Chorin et al. (69)	2020	33,305	Patients divided according to TR severity into none/trace, mild, moderate, and severe	At least moderate TR associated with increased overall mortality in the proportional hazard methods adjusted for clinical and echocardiographic (included systolic function) parameters	Largest evidence on the prognostic role of at least moderate TR, assessed with semi-quantitative guidelines methods
Neuhold et al. (70)	2013	576	Patients divided according to TR severity (significant or non-significant), LV systolic function (mild, moderately, and severely depressed), and NTproBNP levels (below and above the median)	TR associated with the combined endpoint of death, hear transplantation and LVAD implantation only in patients with mild or moderately LV systolic dysfunction and NTproBNP values below the median	The prognostic impact of TR on chronic HF may depend upon the severity of HF
Bartko et al. (71)	2019	382	HFrEF (EF < 40%) and TR evaluated by echocardiographic quantitative methods	Significant increase in mortality in patients with a TR VC ≥ 5 mm, EROA ≥ 0.20 cm^2^ and a regurgitant volume ≥20 ml	New thresholds for quantitative echocardiographic measures associated with all-cause mortality
Agricola et al. (72)	2012	373	LV systolic dysfunction (EF < 50%), at least mild FMR, with or without FTR	Moderate to severe FTR independent determinant of HF, overall mortality, and long-term free of all-cause mortality	At least moderate FTR seems to be an independent marker of end-stage myocardial and mitral valve disease
Höke et al. (11)	2014	239	Divided according to the presence or not of significant lead-induced TR (≥2 TR at follow-up post-implantation)	Significant lead-induced TR in patients with a depressed LVEF (<40%) at baseline was associated with increased all-cause mortality	First study to evaluate the impact of significant lead-induced TR on cardiac function and on the long-term prognosis
Messika-Zeitoun et al. (5)	2020	435,679	HF regardless of EF and at least 1 year of medical history	TR, both prevalent and incident, significantly, and independently associated with all-cause mortality, with increased mortality associated with increased TR severity	Unique insights into the role of TR in HF from a very large database coalescing electronic health and claim records from multiple United States sources

*FTR, functional TR; HFrEF, heart failure with reduced ejection fraction; PH, pulmonary hypertension; AF, atrial fibrillation; EROA, effective orifice regurgitant area; VC, vena contracta; LV, left ventricle; NTproBNP, N-terminal prohormone of brain natriuretic peptide; FMR, functional mitral regurgitation*.

Benfari et al. (3), in their analyses from a large cohort of patients suffering from FTR and HF with reduced ejection fraction, showed that higher FTR grade was associated with considerably worse survival at long-term follow up [adjusted hazard ratios (HR) 1.09 (1.01–1.17) for mild FTR, 1.21 (1.11–1.33) for moderate FTR, and 1.57 (1.39–1.78) for severe FTR], independent of LVSD, PH and across all relevant subgroups. Stratifying for HF stages, moderate and severe FTR was more common in patients with HF stages C vs. B, and compared to trivial FTR, was more associated with systolic and diastolic dysfunction (*p* < 0.0001 for all), confirming its role as a marker of advanced myocardial disease.

In a cohort of patients with LVSD and FTR assessed quantitatively by Topilsky et al. (68), severe FTR (effective regurgitant orifice >0.4 cm^2^) resulted in an increased mortality [HR 1.8 (1.16–2.8), *p* = 0.009], and cardiac events (mortality, new AF or HF) [HR 2.2 (1.1–4.6), *p* = 0.02], both after comprehensive adjustment for age, sex, comorbidity index, LVEF > moderate right ventricular dysfunction, renal dysfunction, AF, left atrium size, and right ventricular systolic pressure. Again, there was a significant link (*p* < 0.001) between TR severity and patients in NYHA class III–IV and with right heart failure.

Chorin et al. (69) have evaluated the role of TR, diagnosed with semi-quantitative echocardiographic methods, in 23,045 patients analyzed retrospectively. One-year mortality rates were 7.7% for patients with no/trivial TR, 16.8% for patients with mild TR, 29.5% for moderate TR, and 45.6% for patients with severe TR (*p* < 0.001). At least moderate TR was associated with an increased overall mortality in the proportional hazard methods [adjusted HR 1.15 for moderate TR (1.02–1.3), *p* = 0.024 and adjusted HR 1.43 for severe TR (1.08–1.88), *p* =0.011] adjusted for age, gender, major comorbidities, and echocardiographic parameters (comprehensive of ejection fraction, PH, and valvular diseases). Ejection fraction and cardiac output were progressively reduced along with the increase of TR severity (*p* < 0.001).

Whether the impact of TR depends on the degree of LVSD is a current matter of debate and was evaluated by Neuhold et al. (70). The authors have demonstrated that the prognostic role of TR may depend upon the stage of HF, as their prospective long-term observational study on 576 consecutive patients revealed that TR was significantly related with the combined endpoint of death/heart transplantation/left ventricular-assist device implantation in patients suffering from mild or moderate LVSD (HR 1.368, CI 1.070–1.748, *p* = 0.0125) but not in those with severe LVSD (ejection fraction < 35%).

Bartko et al. (71) sought to define the natural history of FTR and the prognostic value of its recommended echocardiographic quantification among 372 patients with HF and LVSD. While they confirmed that the severity of FTR increased along with NYHA class (*p* = 0.005) and NT-proBNP levels (*p* < 0.001), surprisingly the thresholds of TR quantitative parameters associated with an increased mortality (*p* < 0.001) were congruent with moderate TR as defined by the present guidelines (73): EROA > 0.2 cm^2^, vena contracta >*5* mm and regurgitant volume >20 ml.

The prognostic role of TR in the context of functional MR and LVSD has been investigated by Agricola et al. (72). Moderate to severe FTR was an independent determinant of HF (HR 1.4, 95% CI 1.1–2.1, *p* = 0.01) and of overall mortality (HR 1.6, 95% CI 1.2–2.1, *p* = 0.01) in 373 consecutive patients with at least mild functional MR regardless of age, PH, RV function or ejection fraction.

It's noteworthy that recent data from the COAPT trial (74), which evaluated the role of Mitraclip in patients affected by FMR, HF and LVSD, enlightened that Mitraclip at 2 years follow-up, compared to medical therapy, improved the composite outcome of death or hospitalization for HF in patients with as well as in those without > moderate FTR. However, in the 98 patients with > moderate FTR, 94 were affected by moderate (2+) TR. These results introduce the concept of a positive effect of left-percutaneous treatment despite the presence of TR, if the latter is not in an advanced stage.

Hoke et al. (11) evaluated the long-term prognostic role of TR following CIEDs implantation: the subgroup analysis of patients with baseline LVEF <40% demonstrated that significant lead-induced TR was associated with poor survival free from all-cause mortality [HR = 2.184 (95% CI 1.112–4.288)].

Indeed, this significant number of studies enlightening the prognostic role of TR seems to justify the changing face of our attention to TR in case of concomitant LVSD. Whether these studies enforce the call for an early intervention is still unclear. In particular, the results provided by Neuhold et al. (70) and Bartko et al. (71) remind us the need for a careful clinical and echocardiographic evaluation of the severity of both HF, and TR before any decision-making: in fact, tricuspid surgery can be done with low-mortality rates only if it is performed before advanced HF stage (75), and we may even not expect the same benefits from percutaneous treatment across all the cohort of these patients.

At last, it is mandatory to underline that TR is a dynamic entity, whose natural course requires a close follow-up, as non-severe TR progression conveys a significant risk of worsening PH, valvular and ventricular remodeling, and long-term augmented mortality (76, 77). In particular, moderate TR in patients with LVSD seem to convey a risk for progression that implies a risk for mortality similar to that of patients with baseline severe TR (76). This is of outmost importance for the surgical management of these patients, as a more aggressive strategy involving TV intervention in patients with mild to moderate FTR and concomitant MV operation has been proven to prevent significant long-term progression of FTR (78).

## Therapeutic Options

In patients with TR and LVSD, the pharmacological therapy aims at two targets: the LV and the hemodynamic consequences of TR. Guidelines medical therapies, such as angiotensin-converting enzyme inhibitors, beta blockers and angiotensin receptor neprilysin inhibitor, may reduce TR, particularly in its early stage, by improving LV systolic and diastolic dysfunction (31, 79). Loop diuretics and mineralocorticoid antagonist reduce TR-induced volume overload and, therefore, decrease PH and systemic venous congestion (31). Nevertheless, medical therapy alone rarely reverts the natural progression of TR in LVSD. Cardiac surgery remains the only definitive treatment, but is rarely performed as in this population, too often too late referred for invasive treatment, is still affected by significant morbidity and mortality (80). Recently, Axtell et al. (81) demonstrated that isolated TV surgery, both repair and replacement, considering surgery as a time-dependent covariate in a propensity-matched sample, was not associated to improve long-term survival compared to medical management alone in a large cohort of 3,276 patients with isolated severe TR. Although single-center and retrospective, with time from severe TR diagnosis to surgical referral varying from 1 to 8 years, this recent study, accounting for remarkable time bias in its analyses, once again underlined the need for optimal timing of intervention in these cohort of patients. LVSD (EF <40%) is a significant independent predictor of mid and long-term more than moderate TR after tricuspid repair, and reoperation for TV carries a significant higher mortality risk (31, 82, 83). Therefore, guidelines clearly designate LVSD as a major determinant of the therapeutical path of these patients, leading the decision, if present, toward a conservative treatment (31).

In the last years, transcatheter strategies, despite being in their initial phase yet, have emerged as a potential therapeutic option. This interventional strategy is of particular interest and need, considering the high-surgical risk typical of the population affected by TR and LVSD. Current treatments include annuloplasty, improved leaflet co-aptation, edge-to edge repair, reduction of the reflux in the vena cava, and percutaneous valve replacement (84–86). The results of the first studies with different devices revealed that transcatheter treatment of moderate and severe FTR is effective in reducing TR severity and, therefore, improving survival and quality of life compared to medical therapy (87–89). Most of the patients enrolled in these studies presented a baseline LVEF > 50%. However, subgroup analyses from the Trivalve registry (89) confirmed the improved outcomes in the 18 patients with LVEF < 35% out of an overall treated population of 472. Conversely, a retrospective study (90) assessing the impact of LV function on the outcomes of TV percuteanous approach, showed that its prognostic effect might be limited to the group of patients with EF > 50%. The authors hypothesized that the pathological hallmarks of HF with preserved EF, diastolic dysfunction and reduced LV filling, may be positively influenced by a correction of TR, as opposed to a compromised LV with systolic impairment.

Up until now, the ideal candidate seems to be a patient with FTR and partially preserved leaflets co-aptation, in the absence of significant apical valve tethering, RV dysfunction and PH (91, 92); in case of extreme left ventricular dysfunction these devices may eventually be considered, in selected cases, only as a palliative approach, but we need randomized clinical trials to address this unmet clinical need.

## Conclusions

Despite being historically recognized only as a surrogate of advanced heart disease, latest studies have clearly demonstrated that at least moderate TR, in combination with LVSD, is a frequent condition, with different etiologies, and associated with independent excess mortality that increases with the degree of TR, highly suggesting of a causal effect that relies on different direct and indirect pathological mechanisms. While we look forward to randomized trials of transcatheter tricuspid devices in a selected population with LVSD, we must continue to improve our awareness of the role of TR in the context of left and right ventricular disease, in order to comprehend whether to consider it a surrogate of advanced myocardial disease or, if a potential target, the most appropriate timing for intervention.

## Author Contributions

All the authors have contributed, read, and approved the final version of the manuscript.

## Conflict of Interest

The authors declare that the research was conducted in the absence of any commercial or financial relationships that could be construed as a potential conflict of interest.
